# Book Review: Supporting Students With Health Conditions

**DOI:** 10.5334/cie.15

**Published:** 2020-03-06

**Authors:** Mindy Elliott

**Affiliations:** 1 Veritas Collaborative; HEAL Association Executive Board, US

**Keywords:** educator resource, pediatric medical conditions, student illness, chronic illness, school issues, hospital students, hospital school, school in treatment, student mental health, homebound students, grief

## Abstract

Review of a practical resource providing educators and clinicians information on specific, common, chronic, medical conditions and insights on how these conditions affect a student’s education.

## Review of *Pediatric Health Conditions in Schools: A Clinician’s Guide for Working With Children, Families, and Educators*

Edited by Allison G. Dempsey, PhD

With an increase of chronic health conditions among children today, many education professionals are finding themselves without the knowledge and experience to understand the medical conditions and how they affect students. *Pediatric Health Conditions in Schools: A Clinician’s Guide for Working With Children, Families, and Educators* (Dempsey, 2019) is a practical and well-organized resource that provides educators and clinicians with information about common chronic medical conditions and insights into how these conditions can affect a student’s education. The text presents case studies to illustrate the complexities associated with maintaining academic progress while facing the physical, social, emotional, and logistical challenges of a student’s illness. Multiple reproducible handouts, such as self-care and pain logs, observation templates, and relaxation strategies, will benefit education professionals in their daily work with these students.

Edited by Allison G. Dempsey, PhD, the book is comprised of chapters written by over 70 experts in their respective fields. Dr. Dempsey is a licensed psychologist working with families and children with complex medical needs in the fetal, neonatal, and early childhood periods. She is an Associate Professor of Psychiatry at the University of Colorado School of Medicine and the Director of Behavioral Health Programs in the Colorado Fetal Care Center and Neonatal Intensive Care Unit at Children’s Hospital Colorado. She has extensive experience working with children who have chronic health conditions from early childhood to young adulthood.

The guide focuses specifically on physical conditions; however, the first two sections provide extensive information on school issues related to any chronic condition, whether physical or mental/emotional. The first chapter notes that other texts, such as one written by McCabe and Shaw (2010), cover specific information in-depth on emotional and mental illnesses.

The book is divided into three sections. Section I focuses on general school-related issues for children with chronic health conditions, including basic medical terminology, social and emotional challenges, and legal issues. Tables are used to organize information allowing for quick reference, and case studies are helpful for understanding the complexities and nuances of maneuvering the school setting when a student lives with a chronic condition.

Section II targets prevention, assessment, intervention, and consultation strategies for individual students as well as school systems. Comprehensive intervention issues include reintegrating students into the school setting after a prolonged absence or a change in their medical status. How to help students deal with grief is also addressed.

Section III concentrates on specific conditions affecting students. Eleven conditions are covered in separate chapters, including diabetes, HIV, and neurodevelopment, pulmonary, cardiovascular, oncologic, and eating disorders. Each chapter starts with an overview of a given condition, followed by social, emotional, and behavioral interventions as well as risk factors and cultural considerations. School and clinical personnel will find the range and depth of information significant, and the handouts and practical strategies particularly useful.

The book highlights the unique situations school personnel encounter when working with students handling a new diagnosis, undergoing treatment, returning from treatment, living with a chronic condition, and processing grief. Educators are often forgotten stakeholders within a student’s treatment process; this text equips school personnel with practical resources and relevant information when faced with student illness.

## References

[1] Dempsey, A. G. (2019). Pediatric health conditions in schools: A clinician’s guide for working with children, families, and educators. New York, NY: Oxford University Press. DOI: 10.1093/med-psych/9780190687281.001.0001

[2] McCabe, P. C., & Shaw, S. R. (2010). Pediatric disorders: Current topics and interventions for educators. Thousand Oaks, CA: Corwin Press.

